# Perspectives on facilitating dynamic ecology courses online using active learning

**DOI:** 10.1002/ece3.6953

**Published:** 2020-11-19

**Authors:** Megan K. Gahl, Allison Gale, Antonia Kaestner, Anda Yoshina, Erin Paglione, Gal Bergman

**Affiliations:** ^1^ College of Natural Sciences Minerva Schools at KGI San Francisco CA USA; ^2^ Minerva Project San Francisco CA USA; ^3^ College of Social Sciences Minerva Schools at KGI San Francisco CA USA; ^4^ College of Arts and Humanities Minerva Schools at KGI San Francisco CA USA

**Keywords:** active learning, case study, course design, education, online learning, pedagogy

## Abstract

As education methodology has grown to incorporate online learning, disciplines with a field component, like ecology, may find themselves sidelined in this transition. In response to challenges posed by moving classes online, previous studies have assessed whether an online environment can be effective for student learning. This work has found that active learning structures, which maximize information processing and require critical thinking, best support student learning. All too commonly, online and active learning are perceived as mutually exclusive. We argue the success of online learning requires facilitating active learning in online spaces. To highlight this intersection in practice, we use a case study of an online, active, and synchronous ecology and conservation biology course from the College of Natural Sciences at Minerva Schools at KGI. We use our perspectives as curriculum designers, instructors, and students of this course to offer recommendations for creating active online ecology courses. Key components to effective course design and implementation are as follows: facilitating critical “thinking like a scientist”, integrating open‐ended assignments into class discussion, and creating active in‐class dialogues by minimizing lecturing. Based on our experience, we suggest that by employing active learning strategies, the future of ecology in higher education is not inhibited, but in fact supported, by opportunities for learning online.

## Graphical abstract

We summarize previous work supporting active learning and present recommendations for facilitating effective active learning in online spaces using our online ecology course as a case study. As a team of students, faculty, and course designers, we offer a unique, collaborative perspective on the implementation of active, online ecology learning. We highlight that as ecology courses transition online both in the immediate future and as technology continues to evolve, employing active learning strategies are essential for providing effective learning.

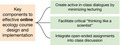

## 1 INTRODUCTION

Though once associated with a lack of academic rigor, online education has become more popular in recent years. Digitization has enabled online learning options to increase access to a more flexible education, and new technologies have enhanced the online learning experience. Higher education, which just this year has been almost entirely relegated to online platforms, is facing an immediate future that is likely to see a majority of classes online, and perhaps a long‐term future where at least a subset remain as such. Students, instructors, and technology must adapt accordingly.

Although a shift toward online education or distance learning may prove to be more convenient or accessible, it presents challenges. Both students and instructors identify the lack of in‐person interaction as a hurdle in learning and teaching (Jaggars, 2014). Discourse and debate are important parts of the learning process (Ni, 2013); online learning, especially asynchronous classrooms, may hinder this communicative aspect. Students also hesitate to take online courses, especially science courses, when they deem the subject matter to be too difficult, due to fears that they would have to “teach themselves” (Jaggars, 2014).

University‐level ecology courses often involve a field or lab component, something that is usually absent from an online course. Research on the effectiveness of online replacements for ecology laboratory and field experiences is notably lacking from the current literature and should be investigated further, especially given the current shift to online learning. Research into the effectiveness of online replacements for laboratory and field experiences in other science fields has been focused on student learning of content (e.g., grades, pre‐ and post‐tests). Recent studies have found that online laboratories are as effective as in‐person laboratories in physics, general biology, and physical geography (Darrah et al., 2014; Son, 2016; Stumpf et al., 2008). In some cases, student grades in online laboratory courses exceed those of students taking in‐person laboratories and led to students having more favorable attitudes toward science (Son, 2016). Virtual laboratories are also cost‐ and time‐saving alternatives to in‐person laboratories (Darrah et al., 2014) and may provide accessibility for physically disabled students (Stumpf et al., 2008). These positive results from online laboratories in other fields are encouraging for ecology courses.

Still, there are strong arguments in favor of teaching ecology online. First, a major reason we offer laboratories in ecology is to teach students to make observations and then test hypotheses and draw conclusions. An enormous amount of observational data has been made open‐source and available online; these allow students to practice scientific data analysis with existing rigorous datasets. Second, you can still have students “collect data” in ways that are set up to explore the concept that you are teaching in class, using existing datasets (e.g., Box 2 and Chapter 1 in Gibbs et al., 2011). Third, today's ecologists need to use many new technological and analysis tools, which can be easily taught in an online setting. Finally, students often learn much more about the practice of field research during a research internship, where deeper learning occurs by working with researchers on a larger‐scale project rather than a handful of laboratories or field trips. Because the goal of most ecology laboratories is to practice scientific skills and critical thinking, our view is that these skills should be incorporated into the fabric of the online course and students should be encouraged to engage in field experiences in an internship.

Without field or laboratory work, an ecology course can easily turn into a lecture series. Laboratories are engaging and exciting for students, and it is challenging to maintain this engagement when transferring a course to an online format. A strategy to tackle this is to incorporate active learning. Active learning focuses on critical thinking and cognitive processing (Kosslyn & Nelson, 2017; Styers et al., 2018) and draws on constructivist theory; according to constructivism, knowledge is not simply transmitted (as in memorization) but rather is built, through student interactions with the world and how it works (Hartikainen et al., 2019). Prince (2004) found that all four facets of active learning studied were beneficial; *traditional active learning*, which encompasses any type of student engagement, including the addition of activities into a passive lecture, *collaborative learning*, where students work toward understanding in groups, *cooperative learning*, where students are measured individually but work together toward goals, and *problem‐based learning*, where the introduction of a problem frames the context of the skills being taught.

Active learning is already underrepresented in face‐to‐face classrooms (Stains et al., 2018), and implementing active learning in an online environment is notoriously difficult. Although studies show increased passing rates and increased critical thinking for students in active learning classes (Freeman et al., 2014; Styers et al., 2018), several studies suggest that students receive lower grades and have lower completion rates in the online version of a course compared to the face‐to‐face version (Alpert et al., 2016; Bettinger et al., 2017). Online courses must be designed to be dynamic online—it is not enough to simply use the same activities and teaching methods on an online platform. There is also a learning curve for professors because teaching online is necessarily different from teaching in person. Experience matters; student outcomes generally improve after the third time a professor teaches the course in an online format (Bailey et al., 2018).

We have found that shifting a class online, however, need not be a barrier to engaging students using active learning. Using the case study of an ecology and conservation biology course from the College of Natural Sciences at Minerva Schools at KGI, a university that conducts all classes in active and synchronous online seminars (Kosslyn & Nelson, 2017), the following sections introduce active learning online as a viable option for future ecology courses. We use our upper‐level (concentration) course Keeping Earth Habitable, a survey course of ecology and conservation biology, as an example throughout, because of its success at engaging students and its continued popularity within the College of Natural Sciences. The authors of this paper are course designers, teaching faculty, and students from the course and we have included brief anecdotal overviews of our different perspectives on this course (Box 1 and 2).

**First‐hand perspectives on learning and teaching ecology via online active learning (i.e., “does it actually work?”)**

**Student Perspective**

**Antonia Kaestner**

My greatest takeaway from this course is to have courage. Especially as an undergraduate it is often very intimidating to be confronted with questions and tasks professionals are also wrestling with. The feeling of having nothing to contribute can be inhibiting in getting class work done and participating in professional opportunities, like internships. However, through the support of fellow students and the Professor, I learned that tackling real‐life problems is both humbling and rewarding.

Key for me is the frequent interaction with fellow students in breakouts, in‐class discussions, and through working together on assignments. Engaging in conversation with fellow students helped me build a more diverse perspective on how to approach problems in conservation biology. It is also a lot more fun to talk things through than to only listen. The input of other students in class keeps me engaged and the break out groups leave room to tailor the learning experience to the group members, rather than having to keep up with the entire class.

Frequent interaction in class also makes it possible to have the Professor clarify concepts or tasks. The active learning format also allows for frequent Professor interactions in class, ensuring that everyone is following along and confusions can be clarified, as they come up. Receiving feedback from the Professor on assignment progress in class and in office hours helps in engaging with real‐life problems. It is encouraging to talk through possible approaches and see where reasoning or data are lacking. Bringing part of the assignment to class also enables collective brainstorming and encourages iteration, something that can be hard for me to do on my own.

Overall, working with other students and seeing their ideas positively shaped my thinking, while interactions with the Professor helped me feel confident in the approach I took to the problems we worked on in this course. And as a result, I now feel more comfortable with approaching problems I know do not have a definitive answer and that are the subjects of professionals’ research papers. It takes courage to stand behind your assumptions and calculations but it feels rewarding to know that I can contribute and that I am learning relevant concepts and a scientific way of thinking.

**Professor Perspective**

**Allison Gale**

Ecologists and conservation biologists are, by definition, studying nature. Thus, I admit that the prospect of teaching ecology online initially gave me pause. That said, I now see many advantages. For one, we know that students working in small groups and then sharing out are beneficial for their learning, and yet the practical challenges of moving chairs or rearranging tables, noise distractions with students overhearing other groups, and the general time sink make this less likely to happen in a physical classroom. With online learning software, with one click of a button students can go into “breakout groups” and then reconvene in the main “classroom” seamlessly. Professors can drop in and interact with students in breakout groups to better facilitate later class discussion.

Well‐designed technology can further improve the classroom active learning experience for students by highlighting to professors which students have been quiet, recording classes for later viewing, feedback, and grading, and integrating polling into lesson plans. Especially with our emphasis on dynamic classroom discussions, the ability to “re‐watch” is a critical feature for the professor and students. Both students and the professor can fully engage in what is being said without worrying about taking notes or providing extensive feedback in real time. Professors must be comfortable with a bit of “scripted improvisation”—driving the discussion based on both a general idea of key points to cover while also responding to whatever a student might say in a poll or verbal response, all while staying on time (perhaps the hardest feat of them all)!

In terms of our approach to assignments, assigning open‐ended case studies adds challenges not just for students but for the professor. Students typically are uncomfortable with open‐ended assignments (feeling the usual “what's the right answer?”), so encouragement and hand‐holding are necessary at the start. As the instructor, I found it helpful to emphasize that a key aspect is to clearly explain their approach, as there are multiple approaches that could “work”. Through the case studies, students might see how difficult it is to come up with meaningful “numbers,” and might lose confidence in the approach. That said, policymakers have to generate estimations similar to those in our assignments. So having the students try their hand at calculations that are clearly needed for real‐world policy is very useful. They get a sense for estimations and how much they can vary. They realize very quickly that priorities can greatly affect outcomes. The grading time associated with open‐ended assignments is increased by quite a bit, as each student approaches it slightly differently. In my mind, however, the real learning occurs when they have to develop their approach; increased grading time in exchange for increased learning is a trade I’m willing to make.

**Active learning, critical thinking, and problem‐solving in assignments**

An assignment that both challenges the students and facilitates active learning is our carbon sequestration assignment. Below is an excerpt from the assignment to provide a sense of the active learning, critical thinking, and problem‐solving components.

**Assignment Instructions**

**Case study 3: Sealaska case**

Background: In Southeast Alaska, the native corporation Sealaska is proposing a shift from old‐growth logging to focus on young‐growth (second growth) timber and then to sell a proportion of the “regrowth” as carbon credits (offsets) to polluters in other US states (primarily California). Sealaska proposes to: (a) allow regrowth on previously cut lands and (b) clear cut some old‐growth areas to then reforest and manage them as second‐growth timber stands. The rule of thumb for these second‐growth stands is to leave 20 percent uncut for at least 100 years; this would be staggered over time (i.e., the 20 percent left uncut will be cut in 100 years; 20% of the total forest will be cut every 20 years).

This case study will be completed in two parts.

PART I: Due as preclass work for in‐class discussion.

- Use the background readings provided for class sessions to understand the different sides of this case. Use the “helpful resources” below to explore different methods for estimating carbon sequestration.
- Estimate the potential carbon sequestration of a plot of young growth versus old growth using the rule of thumb that 20% is kept uncut for 100 years (as described above). Note that this is deliberately challenging and there is no "right answer". Constrain your estimations reasonably based on resources you find and be able to justify the choices you made. Simplifying assumptions are expected; just articulate what you assumed and how it affects your outcomes.
- You should estimate carbon sequestration for three scenarios:
  1. Old‐growth forests with no cutting
  2. Young growth from regrowth of older clear cuts
  3. Young growth created by cutting old‐growth stands
- Based on the readings and your estimations, work out a position to two aspects of the Sealaska case: (a) Is a shift to young‐growth timber the right move? Under what constraints? (b) Should Sealaska (and Alaska native corporations) be allowed to sell carbon credits to offset polluters in California and other states in the lower 48?
- Bring to class: Your notes on the three bulleted scenarios, including your position on each of the two aspects of the case. Bring your estimations and how you calculated them in order to share with your peers and get feedback in class.

[Note: Part II is a formal write‐up of the assignment once students have had the opportunity to bring their initial calculations to class and get feedback from their peers and instructor.]

**Instructor Commentary**

**Allison Gale and Megan K. Gahl**

This assignment facilitates active learning in the following ways:

● There is no obvious right answer! Students are required to use outside resources of their choosing, make assumptions, justify their approach, calculate estimations, and then determine their position on a controversial policy case. This leverages the *problem‐based learning* aspect of active learning and nudges students to apply critical thinking skills.
● Dynamic in‐class discussions are a critical part of the process, implementing *collaborative learning*.
● Students make initial calculations on their own, present to their peers and instructor in class, receive feedback on those calculations, and then get to refine their calculations if needed. This mimics the professional scientific process and employs both *traditional active learning and cooperative learning*.

Note that a version of this assignment has been utilized by one of our authors (M.K. Gahl) at a more traditional university with in‐person laboratories. In the in‐person laboratory, the students would physically go to a forest plot, make average measurements of the tree diameters, types, and spacing in that particular forest plot and then extrapolate for a carbon sequestration calculation. While students do get practice with the fieldwork skills of making tree measurements in the physical laboratory version, the time spent in the field does not necessarily add extensive value to the main goals of (a) identifying the relevant variables and assumptions in an estimation and (b) calculating a relevant estimation and applying it to a real‐world policy issue. Instead, in our online version, students are specifically asked to use the types of forest at the locations where carbon credits might actually be issued and to consider the various approaches to these calculations (e.g., how many species to include, per‐tree estimates versus bulk forest averages), which focuses their attention on the critical thinking and problem‐solving skills that are our goal for the course.

## 2 PEDAGOGICAL OVERVIEW

The Minerva approach is to teach skills and concepts and then to use content as examples for cementing those skills and concepts. This allows the course designers to stay focused when designing online sessions, for example, does this part of the activity allow students to practice measuring biodiversity? If not, maybe it is refocused or dropped. For this course, which is a survey of ecology and conservation biology concepts, we introduce these key skills and concepts in the first half of the course and practice using those skills in case studies and in‐class activities. The last half of the course focuses on case studies in different content areas where those same principles, skills, and concepts apply.

Pedagogical techniques that we use to engage students are based on the premise that the instructor is a facilitator and guide. We use no lectures at Minerva, but instead, draw students to consider the concepts more deeply through in‐class activities and discussion. To ensure engagement, we structure activities with the goal that 100% of students will be engaged at least 75% of the time. The facilitation is just as important as the activity structure. A good facilitator will draw students into the discussion by asking challenging application and analysis questions and try to balance talk time among students (note: we use a feature of our platform, Forum, to monitor student talk time, but this can be done in any platform by noting which students have spoken in class using a simple checklist). A key aspect of our pedagogy is student engagement, and therefore, we use cold calling to bring all voices into the conversation. We regularly use polls and small breakout groups to punctuate and drive discussion.

## 3 CASE STUDY: ECOLOGY AND CONSERVATION BIOLOGY COURSE

Key aspects of this course that are effective for engaging students integrate the four facets of active learning identified and studied by Prince (2004): (a) challenging students to problem solve and think like an ecologist (i.e., *traditional active learning*), (b) in‐class active discussion (i.e., *collaborative learning*), and (c) assignments that are tightly integrated into the in‐class experience (both *cooperative* and *problem‐based learning*).

### 3.1 Encouraging thinking like a scientist

To challenge students to practice scientific thinking using *traditional active learning*, a common in‐class activity in this course is to give students a scenario, and then ask them, for example “from what you know about disturbance ecology concepts, what would you predict? How would you test those predictions?” These scenarios are drawn from published ecology studies (e.g., Gonzalez & Chaneton, 2002; Pastro et al., 2011) and after students iterate in small groups on what they would predict (or how they would design a study), figures (or methods) are revealed from the actual paper. Students then analyze those figures and determine whether their predictions were right. The discussion then extends to focus on the strength of the study design, that is, was the study designed such that the results bear strongly on the hypothesis it was designed to test.

For example, for a class on the disturbance ecology of fires, preclass reading focuses on disturbance ecology concepts and fire regimes. In class, we present students with the experimental design of a peer‐reviewed study they have not yet read, Pastro et al. (2011), including figures from the original study. In this before and after controlled experiment, researchers sought to assess differences in the effects of prescribed burns and wildfires on diversity of different assemblages in grasslands in central Australia. We ask students to work together in breakouts to predict a pattern of alpha and beta diversity for plant and lizard assemblages before, after, and one year after the fires. Students discuss the conceptual basis for their predictions after breakouts, and then, we reveal results figures from the Pastro et al. study. Students interpret the results, compare to their predictions, and then make suggestions (if possible) to improve the study design.

This is also a variation of *project‐based learning* because students apply general theories and skills to a specific example. The activity is designed to reflect the process and thinking of ecologists and give students practice with these skills. We find that students often come to the same predictions as the original researchers and have strong and well‐reasoned critiques of the study design.

### 3.2 Facilitating active dialogue within the classroom

A key piece to keeping students engaged in an online class is creating dialogue to employ *collaborative learning*. We create effective dialogue in class by structuring content in an engaging way and using pedagogical techniques from active learning and facilitation (reviewed above in Section 2). Key to structuring engaging content is allocating in‐class time to areas of confusion, disagreement, or situations where there are multiple “correct” answers. This means students read about theories and concepts *before* class to understand the knowns, and then in class, students actively apply these concepts to different content areas.

Ideal concepts to create dialogue are those in which there is some gray area to discuss and tease out confusions. For example, the early part of the course focuses on measurement of populations and communities. The calculations are relatively straightforward and students read about and practice these as preclass work. Students apply those calculations to real data (e.g., spider communities, Chapter 1, Gibbs et al., 2011), and then in class, we use those analyses to make management decisions and recommendations. This spurs many more questions, because now students have to make decisions and assumptions within the calculations that impact their results and the eventual application of those results. Students often realize that the calculations are not as straightforward as they thought before class. Deeper understanding of concepts results from these discussions, as confusions bubble to the surface and are clarified.

Applied examples in ecology and conservation, which often have no single right answer, provide good fodder for in‐class discussion because different priorities drive the most “effective” solution—something useful for students in the “real world”. One example is during our disturbance ecology unit when we explore the effects of dam removal on a river system, which may help increase habitat and connectivity for lotic species and decrease habitat for lentic species. We draw students into the discussion by having them consider different perspectives, which often leads to vigorous discussion. We sometimes structure the activity with externally conceived stakeholders (e.g., a landowner downstream who may be affected by increased unpredictability of flooding, an environmentalist concerned about an endangered lotic species, a group of fisherman who are concerned about loss of bass when dammed lentic areas are released), but many times the students themselves possess very different perspectives on a particular issue (e.g., some are more concerned about native species, others are concerned about flooding regimes and how that affects downstream human habitation). Considering the perspectives of both external and “within‐class” stakeholders adds interest and complexity.

### 3.3 Open‐ended assignments integrated into class discussion

We use assignments as explorations of *problem‐based learning*. Many of our case studies are drawn from Gibbs et al. (2011) and others were developed specifically for this course to focus on real‐world problems and data (e.g., Box 2). To integrate the assignment experience into the class session, students complete analysis for a specific case study as preclass work and then bring it to class for iteration with each other, using structured peer feedback. We then debrief the case study, including polls to clarify intermittent steps in the analysis often followed by a debate among students about the conclusions they drew from their analyses. We outline an excerpt of an assignment from this course in Box 2 to provide an example of the type of ecological problems we use to drive this problem‐based approach and compare it to an in‐person version of the same assignment taught by one of the authors.

Students turn in a final version of the assignments for this course *after* the additional feedback from class. Through this, they are directly exposed to how data is collected and used to address ecological hypotheses and engage in *cooperative learning* as they develop their own data analysis and receive feedback from their peers. Importantly, they are given the opportunity to improve their approach in and outside of class.

### 3.4 Course summary

Within this course, the instructor is not a lecturer but rather facilitates student mastery of skills and concepts. Throughout the course, students apply learned theories and skills to specific examples, and activities are designed to reflect the thinking process of ecologists. Students practice forming predictions, analyzing, and evaluating studies using, for example, a 'reveal' exercise where they test their predictions against empirical data. Assignments are tightly woven into the class curriculum, and often students discuss their initial analysis of the assignment in class in a structured manner before the final submission. A key to keeping students engaged in an online setting is creating dialogue. Students must consider multiple constraints that affect real‐world ecologists and use online resources to tackle environmental issues. Taking advantage of the principles of active learning and creating a more engaging and fruitful learning environment helps prepare students for future pursuits.

## 4 RECOMMENDATIONS

From our experience in designing, teaching, and learning in this course online, we propose that ecology courses online can be as effective as in‐person classes if a few key best practices are implemented:

● Online learning must become active to effectively support students’ learning and engage students so they process information leading to increased learning.
● Aspiring ecologists need to practice making observations, analyzing data, testing hypotheses, designing effective research studies and inferring conclusions from the results. Most of this can be done in active online active learning classes.
● In seminar‐based active learning, the professor should shift roles from a lecturer to a facilitator to maximize students’ engagement.
● Students can be engaged using tools such as structured activities and discussion, collaborative learning, cooperative learning, and problem‐based learning.

These recommendations are based on our experience in an effective and popular online ecology course. More research focused on the effectiveness of active learning approaches in ecology courses is warranted, especially in the current shift to online learning.

## 5 CONCLUSIONS

We have used our upper‐level course Keeping Earth Habitable as an example of an effective online ecology and conservation biology course using active learning practices. Given the increased accessibility and flexibility, online education is increasingly in demand, yet online education does not always support student learning; both teachers and students are aware of its limitations and worry about enrolling in online science courses (Jaggars, 2014). While structured lab sessions are often absent in online learning, practicing scientific skills and critical thinking is arguably as important as those experiences. For online learning to be effective, educators must strive to design courses that incorporate the principles of active learning and implement relevant teaching strategies that focus on practicing scientific skills, critical thinking, and problem‐solving.

## CONFLICT OF INTEREST

The authors declare no conflict of interest.

## AUTHOR CONTRIBUTION

**Megan K Gahl:** Conceptualization (equal); Writing‐original draft (equal); Writing‐review & editing (lead). **Allison Gale:** Conceptualization (equal); Writing‐original draft (equal); Writing‐review & editing (supporting). **Antonia Kaestner:** Conceptualization (equal); Writing‐original draft (equal); Writing‐review & editing (supporting). **Anda Yoshina:** Conceptualization (equal); Writing‐original draft (equal); Writing‐review & editing (supporting). **Erin Paglione:** Conceptualization (equal); Writing‐original draft (equal); Writing‐review & editing (supporting). **Gal Bergman:** Conceptualization (equal); Writing‐original draft (equal); Writing‐review & editing (supporting).

## Data Availability

No research data were referenced for this paper. Any course data are included within the manuscript.
